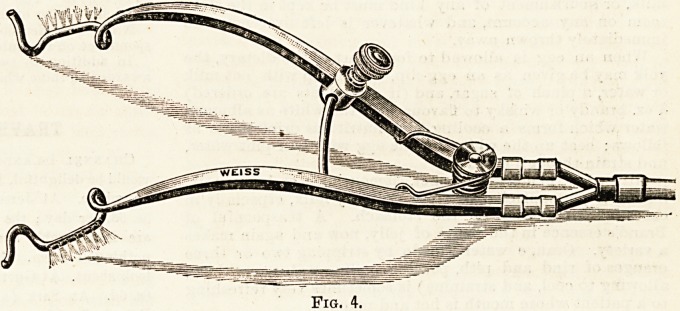# The Hospital. Nursing Section

**Published:** 1903-05-02

**Authors:** 


### The Hospital.

### Nursing Section.

Contributions for this Section of "THE HOSPITAL" should be addressed to the EDITOR, "THE HOSPITAL" NURSING SECTION, 28 & 29 Southampton Street, Strand, London, W.C.

No. 866.—VOL. XXXIV. *SATURDAY, MAY 2*, 1903.

### Notes on News from the Nursing World.

### ROYAL BRITISH NURSES' ASSOCIATION.

PRINCESS CHRISTIAN presided at the General Council meeting of the Royal British Nurses' Association on Friday. The hon. secretary, Dr. Comyns Berkeley, stated that the sum of £36 had been received during the quarter towards the Nurses' Settlement Fund; and that the total now invested was £2,418. He also announced that 27 nurses had been registered; that 29 had been elected members; eight had withdrawn; and two had died. In conclusion, he said that a branch of the Association was about to be formed in Cape Colony. Princess Christian, alluding to the resignation of Miss G. A. Leigh, expressed the thanks of the Council for her devotion to the interests of the Association during the years she had held office. It was intimated that at the annual meeting in June, at which Princess Christian hopes to be present, the subject of State registration for nurses will be brought forward by Miss James.

### BADGES FOR LADY ROBERTS' NURSES.

VERY pretty and unique badges have been presented to Lady Roberts' nurses, who are serving in India, designed by Lady Aileen Roberts, the elder daughter of the Commander-in-Chief. The badge presented to the lady superintendent is of gold, and those to the sisters are of silver. The design is a St. Andrew's Cross within a circle, with bars according to length of service.

### LADY LONDONDERRY ON DISTRICT NURSING.

THE Marchioness of Londonderry presided at the tenth annual meeting of the Newtownards District Nursing Society. Mr. Lavery, who read the report, announced that the balance to the credit of the organisation was £76 15s. 7d., as compared with £61 6s. 5d. last year, and that £1 had been received from a working man as a contribution for the careful attention the society had bestowed upon his deceased wife. Lady Londonderry, in moving the adoption of the report, observed that the contribution mentioned was one of the best testimonials they could have as to the value of the district nurse in the town. In the course of her speech she referred to the maternity cases, of which, to her regret, she only noticed four in the report. She strongly advocated that confinement cases should be taken up by the district nurse. It was, in her opinion, a matter of importance both to the present and future generation, and she urged the committee to consider it. From her connection with several other nursing societies she was able to say that the district nurses very often undertook these cases and saw that the mother and child were looked after.

### THE SECRETARY OF THE CENTRAL MIDWIVES' BOARD.

THE first secretary of the Central Midwives' Board is Mr. G. W. Duncan, of Lamb Buildings, Temple. Mr. Duncan was junior student of Christ Church, and is a B.A. of Oxford. In future, all communications for the Board should be addressed to the Secretary, at the Privy Council Office, Whitehall, S.W., which, by permission of the Lord President, will be at the disposal of the Board until they have decided upon a permanent office.

### THE OUTCRY AGAINST OUTDOOR UNIFORM.

SOME importance has been given to the outcry against outdoor uniform by the statement in the columns of a daily newspaper that the matron of one of the great London hospitals consider it is "a curse to the nursing profession." She justifies this very decided pronouncement on the ground that the nurse's uniform has so long been abused both by those who are professionally, and those who are morally unfit, to rank as nurses; and she further expresses her belief that while "a great many genuine nurses only wear the costume out of laziness, because it is easily put on and thrown off, there are many more who simply wear it to give them an appearance attractive to men." This may be so, and there can be no question that incompetent women often don the uniform in the hope that it may help them to palm themselves off as experienced nurses. But it does not at all follow that, because of its abuse, outdoor uniform should be discarded. Whether on the score of economy, of neatness, or of the saving of time, its use is not only justifiable but desirable. It is seldom compulsory, except among members of the Government services, and so long as most hospital authorities are content to allow it to be optional, there can be no reasonable cause for complaint.

### THE MENTAL STRAIN OF NURSING.

AN inquiry held by the Birkenhead coroner concerning the death of Annie Morgan, a nurse recently employed at the Liscard Nursing Institute, whose body, in nurse's uniform, was found on the beach near Rock Ferry pier, elicited the sad fact that the deceased had committed suicide. Miss Morgan, who was 45 years old, had pursued her profession without break, and had enjoyed good health until about fifteen months ago. At that time she was greatly distressed by the death of a patient whom she had been nursing for seven or eight months, and her mind began to fail. During the remainder of 1902 she was unable to follow her occupation, but at the beginning of this year she seemed better and resumed her work. A few weeks ago, on returning from a case, her manner was strange, but the matron of the Liscard Institute said that as she spoke rationally she did not consider it necessary to forcibly detain her. She visited her sister the next day, and was not seen alive again. It is very seldom that a nurse succumbs, as this poor woman evidently did, to the strain imposed upon her by the performance of her duties, but we doubt whether people who are so quick to criticise and find fault with nurses realise that their work is often exceedingly distressing to the mind as well as arduous to the body. That there are not more terrible collapses like that of Miss Morgan points to the conclusion that, generally speaking, matrons training probationers pay adequate attention to their physical equipment for the career they desire to adopt.

### GREAT INCREASE OF WORK AT CARDIFF.

There was an enormous addition in the number of cases attended by the district nurses of Cardiff in 1902. The visits paid last year were 6,304 more than in 1901. Even those paid to parish cases were 1,645 in excess of the previous year, and the Guardians accordingly increased their contribution to the District Nursing Association from £69 to £80. They might in the circumstances have been a little more generous. At present the committee only see their way to augment the staff by the appointment of one more nurse at a cost of £90 per annum, but it is very clear from the continued growth of Cardiff that this will not nearly suffice. However, a pleasant feature at the annual meeting was the announcement that the workmen are coming forward with help through the works organisations and through their friendly and trade societies. This is one direction in which assistance should be looked for. But the well-to-do classes in Cardiff, who also every year become more numerous, have their obligations towards a movement which has admittedly been of benefit to the town.

### UP-COUNTRY NURSING ASSOCIATION.

We learn from the annual report of the Up-Country Nursing Association, which has just reached us, that the committee had to contend with many difficulties during that period. They started the year fettered with a large debt due to scarcity of nurses, and to the illness of one of their staff. When, at the end of six months, the staff had been increased to five, and a liberal response had been made to the appeal for donations circulated at Naini Tal, the prospects seemed brighter; but in July another nurse contracted enteric fever from a patient, and was not convalescent until November 1st. It was only by means of a fancy fête on the flats and a fancy dress ball organised under Lady Luck's auspices at Naini Tal, that a fresh burden of debt was avoided. It is satisfactory to find that the patients speak with gratitude of the skill, kindness, and self-sacrificing care shown by the nurses. In September, October, and November the demand from all parts of the United Provinces and even beyond, was so great that at one time as many as three cases had to be refused in a single day. The committee of the United Provinces branch hope, by the aid of an additional nurse, who has just gone out, and another to be sent out in the autumn by the house committee, to materially extend the scope of their operations in the present year.

### ATCHAM GUARDIANS AND NURSES.

At the annual meeting of the Atcham Board of Guardians last week, after the chairman and vice-chairman had been re-elected and the committees reappointed, the board proceeded to consider the recommendations of the Officers' Committee. The recommendations were that no further appointment of superintendent nurse be made, but that the matron take on the duties with the title of superintendent nurse, and be paid £10 a year extra for performing them; that two charge nurses be appointed at a commencing salary of £30 per annum, rising to £35; that four assistant nurses be appointed at a commencing salary of £25, rising to £30; and that the present probationer nurse serve as one of the assistant nurses. After some discussion they were carried, and it was decided that candidates for appointments should be requested to come before the board, who will have the assistance of Dr. Cureton in making a selection.

### HALF-TRAINED NURSES FOR NORTHAMPTONSHIRE.

At the first general meeting of the Northamptonshire District Nursing Association a gallant attempt was made to place it on a sound basis from the outset. Sir George Gunning in proposing that the Association should only employ fully-trained nurses, spoke strongly against the lower standard which had been adopted by the committee, and both Lady Knightley and Lady Knightley of Fawsley agreed with him. The latter insisted that fully-trained nurses could be supported if seven or eight villages combined to work on a provident basis, and her own successful experience fully justified the assertion. But the majority took the view that "half a loaf was better than none," and that as the Association could not afford to start with an entire staff of fully-trained women, they had better have part of them village nurses. We think that they have made a mistake, and that if they had acted upon Lady Knightley's advice, there would have been no occasion to talk about choosing the "half loaf."

### NURSES' MISSIONARY UNION.

An organisation called the Nurses' Missionary Union has been formed. It aims "to unite as members all trained nurses who intend to become foreign missionaries," and "to unite as associates all nurses who desire to work and pray for the evangelisation of the world." Its distinctive characteristic is that all meetings are held inside hospital and arranged at the discretion of the nursing staff. At least one joint meeting will be held each year for all nurses in the London district for conference, for devotion, and for the transaction of business. Some of the nurses belonging to the leading London hospitals are already members of the union, but the committee desire that nurses in all parts of the kingdom should know of its existence. Application and full information may be obtained from the General Secretary, 23 Grange Road, Ealing.

### "THE DUTY OF BOURNEMOUTH."

We are glad to see from the annual report of the Society for Providing District Nurses for the Sick Poor of Bournemouth, that an appeal a year ago to

Bournemouth to do its duty—which is incorporated in the report—was not altogether unsuccessful. At any rate the receipts for 1902 exceed the expenditure. But beyond this there is no cause for satisfaction, and though an anonymous donation of £50 may be forthcoming again in 1903 from one of the numerous wealthy residents of the thriving watering-place, the Committee do well to press the need of more regular subscribers. The number of cases nursed was 419, and the number of visits paid was 15,287. The net receipts were £420 2s., of which the poor patients themselves contributed £51, and an addition of £80 contributed in the current year would make all the difference. At Bournemouth, as elsewhere, there has been a falling off in the number of offertories usually given to the society.

### MEDALS FOR ASYLUM WORKERS.

The Executive Committee of the Asylum Workers' Association, whose annual meeting will be held on Thursday, May 14th, have awarded the gold and silver medals for the year 1903. The recipient of the gold medal for attendants is Mr. C. H. Marshall, head attendant at the Eastern Counties Asylum at Colchester, after a service of 48½ years under the same management; while the medal for nurses falls to Miss M. A. Buckle, who has been head nurse in the East Sussex County Asylum for over 43 years. Nine candidates with over 35 years' service—the minimum time qualifying recognition for "long and meritorious nursing service"—sent in applications. The attendant receiving the silver medal, for which there were 10 applications, was Mr. J. Lynch, who has served 33¼ years at the Cork District Asylum; and the nurse, Miss A. E. Jackson, charge nurse, who has been 32¼ years in continuous service at Bethlem Royal Hospital, London. The minimum period of service qualifying for the award of the silver medal is 30 years.

### A NURSES' HOME FOR DUDLEY.

In memory of the late Dr. J. P. Badley, his family have established in Bourne Street, Dudley, a home for four or five trained nurses, who will be employed to attend the sick poor in their own dwellings. Dr. Badley was well known in his lifetime as a philanthropist; his surgery at certain hours of the day was open gratuitously to anyone who cared to consult him, and his advice was much sought and appreciated. The nurses' home will be both a suitable and a valuable memorial of his labours of love in Dudley and the Black Country where his name will long be cherished.

### LIBEL ACTION BY A NURSE.

Mr. Justice Grantham and a special jury were occupied on Friday and Monday last in hearing an action for damages for libel, brought by Miss Alice Jane Beatty, against Messrs. Longmans, Green and Co., the well-known publishers. The alleged libel was contained in the *Month*, a Roman Catholic magazine, and the plaintiff, who said that she had been a nurse for about 16 years, stated that she had been confined in a convent at Chiswick, and only succeeded in making her escape by refusing to take food. She ultimately left in her night-dress and her nurse's cloak. An account of her experiences, which she set forth in a lecture, appeared in the *Rock*, and the *Month* commented upon it, the comments constituting the alleged libel. After Mr. Justice Grantham had addressed the jury, they returned a verdict for the defendants, who did not ask for costs.

### DISTRICT NURSES AT HULL.

The work of the Hull Jubilee District Nursing Association is restricted to one purpose, namely, the nursing of the sick poor free of charge, though contributions towards its funds are invited from those who are able to contribute a trifle. Last year these contributions exceeded £25. The association having wisely refrained from complicating the situation by the formation of a paying branch, have been able not only to maintain their staff of nurses, but also to make an addition. The public in Hull are under no misconception about the 27,410 visits to 790 cases which were paid in 1902. They know that the cost of nursing them must practically be met by their contributions, and they support the charity because of its inherent excellence.

### MOTOR-CARS AND THE HOSPITALS.

Since a nurse and three patients at the London Hospital were taken for a jaunt in a motor-car by a driver who declined to state the name of its owner, Miss Annesley Kenealy has written an article offering to organise a motor-car lending league for taking convalescent women and children in London hospitals for trips. Providing that they are accompanied by a nurse, and that the various hospital authorities are fully informed as to the ownership of the vehicle placed at their service, the scheme is an excellent one. Miss Kenealy recalls the pleasure which was afforded when she was at the hospital for Sick Children in Great Ormond Street by two doctors who used to lend their carriages for an hour or two's airing for the little patients and one of the nurses. There is no doubt that a drive in a motor-car, being more of a novelty, would be still more appreciated, and we hope that the league which Miss Kenealy desires to form will come into existence while the days are long, and the most enjoyment possible can be obtained by those whose lot it is intended to brighten.

### THE M.A.B. AND AMBULANCE NURSES.

At the meeting of the Metropolitan Asylums Board on Saturday a letter was read from the Local Government Board, forwarding for the consideration of the managers an extract from a report by their architectural department on the plans for additional accommodation for ambulance nurses at the South-eastern Ambulance Station, and stating that they were not prepared to sanction the present proposal on the ground that the cost appeared to be disproportionate to the amount of accommodation which would be obtained. The letter was referred to the Works and Ambulance Committee for consideration.

### SHORT ITEMS.

The s.s. *Saxon*, which arrived at Southampton on Saturday, had on board Nursing Sisters E. Townsend and A. L. Pierce, of the Army Nursing Service Reserve.—At an examination held for junior probationers in the West Ham and East London Hospital, Stratford, Nurse Thompson gained the first prize and Nurse Newton the second.

### The Nursing Outlook.

"From magnanimity, all fear above;
From nobler recompense, above applause,
Which owes to man's short outlook all its charm."

### THE SCHOOL NURSE.

When the present School Board of London is swept away by the new Education Bill, there is one of its good works which we sincerely hope may remain and prosper, and that is the visiting nurse for elementary schools. To those who are familiar with fever hospitals and know how the wards are ever filled with children, and to those who have had to nurse through an epidemic of diphtheria, there is no need to dilate on the school as a centre of infection. These big ills have been kept vividly forwarded by the annual reports of Dr. Shirley Murphy and others; but there is another side which appeals chiefly to the district nurse and to the social student, and that is the spread of nasty little complaints such as pediculosis is in schools. Many a mother would gladly take advantage of the excellent teaching in some of the schools were she not afraid that her children would come home with a "dirty head," or with "sore eyes," or with some sort of vermin infesting the clothes. There is no more terrible evil to be faced in slum work than the presence of vermin in enormous quantities in the houses and on the persons of the inhabitants; and great though the risk of diphtheria may be, the general deterioration of health due to dirt can never be ignored. The teachers in some of the Board and Voluntary schools who had girls in their charge reported that 90 per cent. of their scholars suffered from dirty heads; and others reported that "sore eyes" were never entirely absent from their classes, while outburst of "sores round the mouth" were very frequent. A voluntary body some seven years ago had started one or two nurses to visit some of the poorest schools, and to examine the children and give them hints as regards cleanliness, and where necessary to actively interfere to prevent the spread of contagion; but vermin is not a pleasant subject of conversation, nor a subject that appeals to the rich giver, and the London School Nurses' Society was never rich enough to supply more than three or four nurses to London's over 500 schools. But when the question of ringworm was raised the School Board was induced out of the rates to appoint one nurse to report on the prevalence of the disease, and subsequently to appoint two others to aid her. The voluntary nurses continued their work, but the Board's own nurses reported to its medical officer, and their efforts obtained publicity, and really it had seemed of late as though compulsory education might in time not necessarily mean compulsory contamination with some low form of infection.

The work of prevention is ever apt to be less interesting than the work of cure; it probably sounds very dull to a nurse to go round visiting Board schools and cleaning dirty heads and dressing broken chilblains, and perhaps twice in a week detecting a case of mumps, or diphtheria, or scarlet fever, and warning the teacher. There are no operations, none of the fellowship and stir of hospital life, and none of the fierce struggle against the near approach of death. But we believe that in the lessons of cleanliness and the possibilities of cleanliness, and in the stay of the spread of infection, the school nurse does a work for London which makes her a worthy and a noble citizen. And especially is there praise due to those who took it up in its early days, when the work was poorly paid, the teachers had to be propitiated for the extra trouble, and the anxious mothers and fearful children had to be met with tact and forbearance and patience. To the credit of the first school nurses be it recorded that they found welcome everywhere, and never once got at cross-purposes with the powers that be, and that above all they won the affection and the confidence of their little patients. Children are often very sensitive, and the lack of cleanliness, which is not their fault, is often very humiliating to them. Many of these little ones were intensely grateful for the freedom from vermin obtained, and for the nurses' simple directions which taught them how to maintain cleanliness of person. So far as true elevation goes, we are not sure that the teaching of the nurses did not far outweigh the teaching of the teachers, and therefore it is that we hope that the new education authority will see to it that the school nurse has her place in the new system. Take the question of contagious eye disease alone: what use is it to teach a child to read and sew if meanwhile you allow it to contract a disease which must end in permanent blindness? Surely the ratepayers have a right to demand that the school nurse shall continue.

In Germany the school nurse is an old nstitution, and in Liverpool and other English towns she is now at work. In New York, as in London, she was first set afoot by a voluntary agency. There is daily medical inspection of schools in New York; but the doctors have only the right to exclude; they do not treat the children, nor follow them home to see what becomes of them. Such a system was of purely negative value, and one medical man got a nurse to follow up certain cases he told her about, and see that the parents tackled the question of vermin, or took the child to an eye hospital. So good was the result that the Board of Education lately appointed nine nurses of their own to work under them, and at a sweep New York went ahead of London on this subject. But we shall catch up again soon. Let all who have influence bring it to bear on the new authority when created, and London also may boast a sufficient supply of visiting nurses to teach elementary cleanliness to the citizens of the future.

### Lectures on Ophthalmic Nursing.

By A. S. Cobbledick, M.D., B.S.Lond., Senior Clinical Assistant Royal Eye Hospital, late House-Surgeon and Registrar, Royal Eye Hospital.

### LECTURE IX.—DRESSINGS (*cont.*).—INSTRUMENTS IN COMMON USE.—THEIR STERILISATON.

The dressing which is applied next to the lid must be aseptic. Of course the lids themselves, provided the conjunctival sac is quite healthy, form an excellent support or splint in all operation cases; if by any chance the sac is unhealthy from conjunctivitis or a discharge regurgitating from the lachrymal sac through the puncta, then the efficacy of the dressing is lost. The pad and bandage serve two purposes: firstly, to keep the lid from opening; secondly, they exert a uniform and elastic pressure on the eyeball.

Fig. 1.
Steam Sterilisers for Dressings, Bandages, Operation Cloths, etc.

*Preparation of Pads.*—They are made of white absorbent wool. Provided one of the guards prepared as above is used, the pads need not be sterilised; every care, however, should be taken to prevent the wool from being in any way infected; they should be cut out on a clean mackintosh and the scissors should be immersed for a few minutes in boiling water previous to use. When the pads are cut it is usual to "bake" them in an oven: this process causes them to swell, so that when applied to the eye more elastic pressure can be exerted by the bandage. After undergoing this process they should be kept in an air-tight japanned tin dressing-box. Where an aseptic guard is not used, the dressing next the eye, whether wool, lint, or gauze, should be sterilised by steam. The following diagram represents the dressing steriliser in general use.

*Directions for Use of Sterilising Apparatus.*—1. First fill the outer jacket or boiler with water about one-third full, through the funnel (A). The height of the water is readily ascertained by means of the water gauge (B).

2. The materials to be sterilised may then either be put directly into the body of the apparatus, or may be first placed inside suitable containers, such as Schimmelbusch's kettles; but if containers are used they must be so constructed as to allow free passage for the steam. A layer of absorbent wool should be placed over the top to catch any drops of condensed steam from the lid.

3. The lid is then replaced and fastened down firmly by means of the screw nuts.

4. The burner underneath the apparatus is then lighted, and the escape tube (C) carried into the condenser (D); a few inches of water should be placed in the condenser just sufficient to cover the end of the tube.

The materials should be left subjected to the steam for 30 minutes after the thermometer reaches 100° centigrade.

Before putting out the lamp the tube should be removed from the condenser. The lid may now be opened, and when all the steam has escaped the layer of wool may be removed and the dressings will be found ready for use.

N.B.—The steam enters the sterilising chamber at the

Fig. 2.
Bronner's Steriliser, for Eye Instruments, Dressings, and Solutions. The instruments are boiled in soda solution, and the dressings subjected to steam.

top, and is kept at the same temperature on its way downwards by the heat of the outer chamber. The materials will be found comparatively dry when removed; therefore no special drying arrangement is required.

Bronner's steriliser for eye dressings and instruments is very convenient and useful.

*Description of Apparatus.*—A, The body of the apparatus or boiler. B is the gas-cock, and C the opening through which it is lighted. D, Tray for containing eye knives, forceps, and scissors. E is the upper part of apparatus, or steamer, for bandages and solutions. F and G, Wire baskets for containing bandages or dressings (G fits on the top of F). H, Stand for bottles of solution. J, Cover, which can be fitted either on to the top of E or of A, as may be desired. K, Escape in cover for waste steam. L shows the complete apparatus; MMMM, the catches by which the various parts are bolted together; and NN, the handles for carriage.

*Bandages* should be 2 inches in width, of bleached calico, openwove, or flannel. Liebreich's bandage is useful in cataract cases, where both eyes are closed for the first four days. It consists of a wide oblong piece of open wove material at the corners of which are attached four pieces of wide tape.

*The Care of Instruments.*—Though the nurse in hospital has but little to do with the care of instruments, excepting in the out-patient department, it is important that she should be able to meet an emergency and know the names of most of the ophthalmic instruments in daily use.

*The Eye Speculum.*—A spring contrivance to keep the lids widely open during operations on the globe of the eye. Figs. 3 and 4 represent two of the most useful forms.

In both these specula it will be seen that when the lids are sufficiently wide open the arms can be fixed by a screw.

Webster Fox's (Fig. 3) has the advantage of a hinge in each arm, so as to allow the weight of the speculum to rest on the temple, even when the eyes are deeply set.

Fig. 3.

Fig. 4.

Fig. 4 is a device by Messrs. Weiss and Son whereby the speculum and douche are combined. There are many other forms of eye speculum, all working on the same principle.

### Liquid Diet for Typhoid Patients.

### EXAMINATION QUESTIONS FOR NURSES.

The question was as follows:—How would you feed a patient suffering from enteric fever whilst the nourishment ordered by the doctor is to be strictly liquid? N B—The question supposes that the medical man in attendance gives only general orders and leaves the nurse to exercise her discretion.

First Prize.

The diet in a case of typhoid fever is one of the most important points, and while on liquids, it is left to the nurse to vary as much as possible the necessarily limited diet. The patient is usually kept on liquid food till the temperature has been at normal for a week or ten days. Milk, of course, is the most suitable food. If this is used alone, three pints at least should be given to an adult in the 24 hours, always diluted with water, lime or barley-water, or soda-water. The stools of a patient on milk diet should be examined with great care to see if the milk be entirely digested. If masses of curds appear the milk should be partially peptonised, or it may be varied with beef-tea, or chicken or mutton broth. Eggs may sometimes be given beaten up in milk, or, better still, in the form of albumen-water. To prepare this, strain the whites of eggs through a cloth and mix with an equal quantity of water. A flavour of lemon greatly improves this, and if the patient is taking stimulants it can be very conveniently given with this. Some patients are unable to take milk, but they can subsist for some time on this alone. Sometimes they will take whey or butter-milk when the ordinary milk is distasteful. The patient should be encouraged to drink water freely which may be given pleasantly cold. Iced tea, barley-water, or lemonade may also be given, and as a rule there is no objection to a moderate quantity of cocoa or coffee.

Typhoid patients should be fed at regular intervals through the day. It is better to give them a small quantity frequently. It depends upon the condition of the patient whether or not he should be roused from sleep for nourishment. In mild cases it is advisable not to disturb him, but where there is stupor he should be roused for food at regular intervals night and day. Mab.

Second Prize.

Milk is looked upon as the staple article of diet in enteric, and the patient should take from two to three pints of it in the twenty-four hours, as well as one pint of beef or chicken tea. The milk should in all severe cases be diluted, either with pure water (in the proportion of about one part water

### LIQUID DIET FOR TYPHOID PATIENTS—*Continued.*

to three parts milk); or soda-water, lime-water, or barley-water may be added if liked better; the former should not be given if there is a tendency to tympanites. When digestion is weak, the milk or beef-tea is better given peptonised; the "Zymine" peptonising powders are simple to use, and full directions are given with each packet. The utmost care should be taken in the preparation of the beef or chicken tea; it must be free from the smallest suspicion of grease, carefully strained, and the too-often forgotten pinch of salt added before serving. Should there be an inclination to diarrhœa, all beef-tea or chicken-tea must be discontinued for some hours, or a large pinch of isinglass dissolved in it while boiling may have the desired effect.

A small cup of tea may be given morning and evening if the patient likes it; it will generally be looked upon as a welcome variety from the milk; care must, however, be taken to see that it is freshly made with boiling water and poured into the cup through a strainer to prevent all danger of stray tea-leaves. The prospect of a "nice cup of tea" will often induce a refractory patient to take his or her ordinary nourishment when other persuasions have been in vain.

A supply of pure boiled or filtered cold water ought always to be at hand, as a drink at intervals will be found most grateful in relieving the intense thirst; also a good supply of ice broken into small fragments which the patient can suck when desired. Needless to say, however, no water, milk, or nourishment of any kind must be kept in the sick-room on any account, and whatever is left over must be immediately thrown away.

When an egg is allowed to form part of the dietary, the yolk may be given as an egg-flip, beaten up with hot milk or water, a pinch of sugar, and (if stimulants are ordered) ½ oz. brandy or whisky to flavour, and the white as albumen water which forms a cooling and nutritious drink made as follows: beat up the white of one egg with half-pint water, and strain through muslin.

Milk whey, made with rennet, lemon-juice or buttermilk, makes a pleasant change from ordinary milk, especially in cases of irritability of the stomach. A teaspoonful of Brand's essence in the shape of jelly, now and again makes a variety. Orange water (made by stripping two or three oranges of rind and pith, pouring boiling water over them, allowing to cool, and straining) is sometimes very refreshing to a patient whose mouth is hot and parched.

I have said nothing about stimulants, as their nature and amount will, of course, be strictly prescribed by the physician in attendance.

An enteric patient's diet is necessarily an exceedingly limited one, and he is very apt to tire of the same things over and over again; the great points to remember are to give nourishment "little and often" (every two or three hours is enough in an average case), to serve it daintily, and to vary the routine as much as possible, giving milk alternately with beef-tea, beef-tea with chicken-tea, plain milk with milk whey, etc.; from a few teaspoonfuls to about four or five ounces will probably be as much as will be required at a time. Erin.

### The Virtues and Defects of the Answers.

The two prize papers are long this month. As a whole the answers are good, showing care and thought to do the best for the patient; but there is a great paucity of ideas as to variety. The two prize-winners are successful because they venture upon some slight variety, but they might go much further. Only one nurse mentions Koumiss, a most delightful drink liked by almost all. But there are so many alterations that may be made and yet remain strictly within the regulation "liquid." Creams of all kinds, flavoured to prevent monotony; orange juice with soda water, lemon ditto; plain cream instead of milk, whipped cream flavoured. Moulds of milk and cream flavoured and stiffened with isinglass are usually allowed by the doctors to come under the term liquid, because they dissolve in the mouth and stomach. Veal broth, chicken broth, and especially oyster broth are useful. Valentine's meat-juice does not increase diarrhœa like beef-tea, and is palatable iced. Mixed with hot water it is distinctly nasty.

### Honourable Mention.

This is gained by "H.O.C. Age," "Nancy," "Lilian," and "Beatrice." "Lilian's" answer is very good, but her quantities are too large. Few patients can stand 8 ozs. every two hours.

### Views on the Subject from Beyond the Seas.

Next week we shall see what our fellow nurses in the colonies and abroad generally have to say on the subject.

### Question for May.

What precautions should you take to avoid splint sores in cases of excised elbow joint, and fractured femur?

The Examiner.

### Rules.

The competition is open to all. Answers must not exceed 500 words, and be written on one side of the paper only. The pseudonym, as well as the proper name and address, must be written on the *same* paper, and *not on a separate sheet.* Papers may be sent in for fifteen days only from the day of the publication of the question. All illustrations strictly prohibited. Failure to comply with these rules will disqualify the candidate for competition. Prizes will be awarded for the two best answers. Papers to be sent to "The Editor," with "Examination" written on the left-hand corner of the envelope.

N.B.—The decision of the examiners is final, and no correspondence on the subject can be entertained.

In addition to two prizes honourable mention cards will be awarded to those who have sent in exceptionally good papers.

### TRAVEL NOTES AND QUERIES.

Channel Islands (J. F.).—In an ordinary season the Islands would be delightful, but this year the bitter cold will be unpleasant anywhere. At Jersey the cheapest hotels are the Weigh Bridge, 5s. 6d. per day; the Star, 6s.; and The Grasshopper, 6s. There are innumerable apartments and boarding houses, but I know nothing of them. Go to one of the above hotels for a night and look about. At Guernsney The Crown, 5s. 6d., and the Broughton, 6s. 6d. At Sark (a charming island) not very much choice, the Bel Air, 7s. 6d., and the Dixcart, about the same.

Generalabonnementen System (Viator).—Thanks for your letter. You are right, it *is* a good plan. Most of our correspondents, however, have such short holidays that they don't think it worth while to get one. There is something to be said on that score, but many thanks for your clearly expressed letter.

A Week's Holiday in Brittany (M. A. B. C.).—St. Servan would suit well; in itself it is not pretty, but is an excellent centre for excursions. Second return to St. Malo £2 1s. 2d. Maison Mathias would suit you, or Miss Humphrey, 20 Place Constantine, both 7 francs per day, or less out of the season. Spend your first or last Sunday at Mont St. Michel Hôtel Poulard Ainé. Remember the steamers go only three times a week. You will find full particulars of this part of Brittany in articles for May 6th and May 13, 1899, in The Hospital. A week will cost you, with excursions and tips, about £5, including journey, and 10 days about another 30s.

Holiday in Belgium (St. Patrick).—Yes, you can have your three weeks' holiday, all three of you, for the sum you mention. Cut out Ghent from your programme, because it can be seen easily from Bruges. At Brussels go to Hôtel du Rhin, 14 Rue de Brabant. Ask for rooms on third floor. Pension terms 6 francs. Ask them to take the three of you for 5 francs each. Write beforehand. From Brussels you can visit Louvain-Malines and Alost. Each one can be fairly seen in one day. If you are very energetic you could also manage Antwerp, just seeing the Cathedral, St. Paul's, the Steen Prison, and the picture gallery. After a week or ten days go to Bruges. Send me a stamped and addressed envelope and I will give you a private address there. I prefer Bruges much to Ghent. From there visit Ghent, Courtrai, and Oudenarde. Your journey (to come within your means) must be from Tower Bridge to Ostend, fare return first class 10s. 6d. I hope this is all clear to you, if not write me again. Thanks for kind remarks on my articles about Italy.

### Four Months' Training in a Maternity Hospital.

BY A NURSE IN THE NORTH.

(*Concluded from page* 45.)

### Outside Work.

Now I have come to the part of my experience which I found most interesting—namely, the outside work on the district. The work lay entirely in the poorer part, and some of the houses, or rooms rather, were terrible abodes of poverty, caused in most instances by drink, and the dirty state of everything was deplorable. But in this respect I was fortunate, and I never but once had to wait for long in a dirty house. The patients for the most part were very grateful for the attention they received, and much preferred having a nurse to a student. Indeed, in one case when the people who came to fetch a nurse had been given a card to go for a student, they returned after an interval to say he was not at home, which the night nurse knew could not be true as he had not been called out before during the night; and when she cross-questioned them their story broke down, and they acknowledged they had never been to the house, as they wanted a nurse. Another point which I must notice was the respect invariably shown to nurses in uniform even in the most drunken and vicious neighbourhoods, and the unfailing readiness to help one to find a street displayed by policemen and tram-conductors. The former were my resource in all my difficulties, and even if they had never heard of the place I wanted they would look it out in their street directory, and not be satisfied till I had an idea of its whereabouts. The first time I was taken out it was a false run, and the same luck attended me the next time, although in that instance I returned later in the day and "got my case." At first, a nurse is taken out by a senior, and allowed two cases before she goes by herself; and both of my cases I had with a very nice Irish nurse, who, although she came after I did, had got ahead, owing to my enforced absence. The second case I got with her was on New Year's Day. I had been out in the afternoon with another nurse, but the baby was born before we arrived; the patient and two other women who were in the room with her were in a state of intoxication; on our return I was sent out again to follow the Irish nurse ——, and get the case if I could. The locality was new to me, and, as it was dark and raining, it was with difficulty that I found the street; when I did so I was fortunate enough to find a clean house, and the patient, an Irishwoman, was a very nice woman. After waiting a little I "brought the wean home" all right. After this I lived in hourly expectation of being sent out by myself, which came to pass one morning at 3 A.M. The women who came to fetch me were barefoot, and wore shawls, so I judged the house would be poor; but I was not prepared for the absolute poverty and filth which I found. I did not get the case as I had to send in for the doctor, and he brought two students with him, so I had to return. My first case alone was still to come, and although when it did it was a normal one, I found matters rather difficult as my patient was a Jewess who could not speak English, nor understand very much of it. Altogether I had three Jewesses, so I became an adept at talking by signs.

### The Early Hours of the Morning.

The worst part was being called in the early hours of the morning and having to turn out in the cold. On one occasion I went out about 1 A.M., and as my patient was a primipara and making very little progress, I left her telling them to come for me later on. Snow had fallen and everything looked so white and clear, and where in the daytime all was bustle and noise there was now peace and quietness. It was a phase of city life which is given to few to see and made me think of a line which, I believe, occurs in a poem of Wordsworth, composed upon Westminster Bridge. He imagines himself to be standing on London Bridge in the early hours of the morning: "Dear God! the very houses seem asleep, and all that mighty heart is lying still." On my return, after taking the cup of tea which the night-nurse always made for those who had been out, I returned to bed hoping to finish the night in peace. But alas! no; my patient sent in for me within an hour, and I had to return through the snow, and to crown it all I got my feet wet and had to stay with them in this state for several hours, and in this particular house the people did not think of offering me even a cup of tea, although it was after midday before I was ready to leave. The result was that I got a bad cold and cough, and matron removed me from the district to the wards, to my great sorrow. I had thoroughly enjoyed the visiting, going from house to house in the district which was every morning allotted to each nurse, except it was her turn to wait in for a case, attending to the patient and washing and dressing the baby. I had even been privileged to take out a junior nurse with me. One of the latter instances deserves special mention: the house was situated in a main street and when our conductor opened the door we found ourselves in a naturalist's shop, the walls lined with cages in which were white rabbits, pigeons of every description, and various other birds. The living-room was behind the shop, and as it was only lighted by a window in the roof, the gas was kept burning all day; and in this room lived a man, his wife, and six children. The atmosphere can be better imagined than described. On my reappearance in the wards I found myself on a different footing from when I had left them to go on the district. I was now a senior with the privilege of dealing out the patients' lunch, tea, and supper, breakfast and dinner being served by the staff nurse; and as I went into a ward where one of my contemporaries was in charge, I had not the responsibiiity of management, which is wearisome when the nurses are always changing, and some have not the most elementary knowledge of nursing.

### Lavatory Duty and Lectures.

My cough became less troublesome, but just as I was hoping to return to the district I got a septic finger, and although that healed and I had a few more outside cases, another finger became septic, and I finished my career on "lavatory duty." This included taking all dirty clothes to the laundry, bringing up the clean cloths for the babies, and on Sundays keeping in the laundry fire and drying the babies' cloths after the nurses had washed them. I must just say a word about the lectures. Four visiting-physicians gave these in turn, and some of them were really splendid; but one's attendance was very uncertain owing to the uncertainty of the cases either outside or inside the hospital. In addition, the resident house-surgeon lectured to us either in the lecture-hall or round the patients' beds, and the latter method was also adopted by two of the visiting doctors. Our exam. day was a memorable occasion, or, to speak correctly, I should say days. Three of us went up together and we wrote our papers on a Saturday night, but as it could not be arranged for the oral exam. to take place on the same day, we had to live in suspense over Sunday. The fact that we were not certain of passing did not, however, deter us from giving a "diploma tea" on the Saturday night. The dreaded ordeal was passed at last, our papers signed by the two examining doctors and matron, and we had the proud satisfaction of calling ourselves "certificated midwives." My stay was brief after receiving my certificate, for within two hours I had packed my boxes and left the hospital, and so ended my four months' maternity training.

### Everybody's Opinion.

[Correspondence on all subjects is invited, but we cannot in any way be responsible for the opinions expressed by our correspondents. No communication can be entertained if the name and address of the correspondent are not given as a guarantee of good faith, but not necessarily for publication. All correspondents should write on one side of the paper only.]

### RADCLIFFE INFIRMARY, OXFORD.

"Miss Agnes Watt," matron of the Radcliffe Infirmary, Oxford, writes: May I be allowed to correct a statement which appears in the "Notes and Queries" column of last week's issue of The Hospital? The term of training in the Radcliffe Infirmary is now three years.

### GIFTS TO NURSES.

"N. J. J." writes: As a fellow nurse may I express my appreciation of the way an English nurse gives voice to her opinion of gifts. I fully re-echo her statements and trust that all nurses who accept gifts, receive them not from an avaricious disposition but from a feeling of love and sympathy. A nurse who acts otherwise is not worthy of her high vocation.

### ROYAL NATIONAL PENSION FUND FOR NURSES.

"A Matron of Q.A.I.M.N.S." writes: I was so glad to see a letter in your paper about three weeks ago expressive of grateful thanks to all the gentlemen concerned in the management of the Royal National Pension Fund. Personally, I feel that nurses as a whole never can be sufficiently grateful, both to the founder of the scheme for his thoughtful interest in their welfare, and also to those expert men of business who make time among the many and important affairs claiming their attention to look out the best investments for the savings of the members of the Fund. Being one of those who joined the Fund soon after it was started it has always been my object to induce others to avail themselves of its benefits. I hope that you will find space for this letter in which I feel I have not expressed half the gratitude all nurses ought to entertain for the promoters of the Royal National Pension Fund.

### WORDS OF ADVICE TO NURSES.

"Twelve Years a Private Nurse" writes: May I through your valuable paper thank Miss Esther Young for her excellent "Lectures to Nurses" which have appeared during the past few weeks. Also suggest to "The Hermit Crab" to reread them all very carefully.

"Nurse Katharine" writes: I should like to say how fully I endorse "Hermit Crab's" opinion with regard to nurses having a distinct life and interests outside their profession. Why should a nurse, any more than anyone else, be narrowed down to a groove, with no individuality of her own and no ideas but those pertaining to her profession? In no other profession would such a thing be applauded or even tolerated. Why should it be in the, to my mind, highest profession of all? I believe that to be a really good nurse a love of one's work and a strict attention to duty is absolutely necessary, but not sufficient. Especially is this the case in private nursing. One is sent to nurse intelligent, intellectual men and women, and how deadly wearisome must it be to them in the long hours of convalescence to have for chief companion a woman who, though sympathetic, kind, and a perfect nurse as far as mere nursing goes, cannot converse intelligently on a single subject outside her work, nor interest herself in any of the pursuits which are nearest the patient's heart. I think that every nurse, hospital or private, should, when off duty, endeavour to completely forget her work; she will then return to it with renewed zeal and energy and with a fund of fresh ideas to enliven the weariness of the long hours of her patient's convalescence, and to keep herself from getting cramped and narrow and "groovy." I may be wrong, but that is my idea of a perfect nurse.

### Appointments.

Farnham Private Asylum, Finglas, County Dublin.—Miss Margaret S. Gordon has been appointed matron. She was trained at the Western Infirmary, Glasgow, was after wards engaged for some years in district nursing, and has lately been one of the assistant matrons at the Stirling District Asylum, Larbert.

Goole Urban District Council Fever Hospitals.—Miss Rosalyn Wright has been appointed matron. She was trained at the Borough Hospital, St. Helen's, and has since been staff nurse at the Isolation Hospital, Brynn, and charge nurse at the Goole Sanatorium.

Grantham Hospital.—Miss Maude Sawle has been appointed matron. She was trained at the Cardiff General Infirmary and has since been charge nurse Newport and County Hospital; sister of Children's Medical and Surgical and Women's Accident Wards, Wolverhampton Hospital; and sister-in-charge of male surgical wards with temporary charge of surgical wing and theatre Tobay Hospital, Torquay.

Home for Protestant Incurables, Cork.—Miss E. F. B. Sandeford has been appointed lady superintendent. She was trained at Crumpsall Infirmary, Manchester, and for the past six years has held the post of staff sister and assistant matron at the Home for Protestant Incurables, Cork. She has also had experience in private nursing.

Isolation Hospital, Hertford.—Miss M. Garrood and Miss Lane have been appointed charge nurses. Miss Garrood was trained at Northampton Borough Hospital, and has since been nurse at Leicester and Croydon Borough Hospital. Miss Lane was trained at the Western Fever Hospital, Fulham, and has done six months' private nursing at Southsea.

Royal Bath Hospital, Harrogate. Miss M. H. Marriott has been appointed head nurse, and Miss E. M. Sutcliffe staff nurse. Miss Marriott was trained at St. Mary's Hospital, Paddington, and Miss Sutcliffe at Bradford Union Infirmary.

Sheffield Union Infirmary.—Miss Emily Berry has been appointed night superintendent. She was trained at Sheffield Union Infirmary, and has since been staff nurse and sister in the same institution.

### Presentations.

Birmingham Hospital Saturday Fund.—An interesting ceremony took place on April 20th at the People's Hall, Hurst Street, Birmingham. Miss Duggan, who was for six years matron of the Birmingham Hospital Saturday Fund's Convalescent Home for Women, "Yr Erw," Deganwy, near Llandudno (which has recently been closed), was presented by her old patients with a handsomely-bound illuminated address and a silver Queen Anne tea service. During the evening a beautiful basket of choice flowers was also handed to Miss Duggan.

Cumberland Infirmary.—Miss Mary Armstrong has been presented by the nurses of the Cumberland Infirmary, Carlisle, with a silver sugar basin and cream-jug on a silver salver, together with a pair of sugar-tongs and half-a-dozen afternoon teaspoons, on the occasion of her leaving to take up duties as sister of the children's ward and theatre at the Southport Infirmary.

Perth Royal Infirmary.—Miss Logan, who has been 25 years matron of the County and City Royal Infirmary, Perth, has been presented with the following gifts:—House surgeon, silver flower-vases; nursing staff, easy chair; servants, silver-plated teapot, cream-jug and sugar-basin, and afternoon teacups and saucers.

### Echoes from the Outside World.

### The King in Italy.

THE King, who arrived at Naples on Thursday last week and later in the afternoon visited Queen Amelia of Portugal on board her yacht *Amelia*; the German Crown Prince and his brother on board the *Sapphire*; and the Duke of Abruzzi on board the *Liguria*. Next day Lord Rosebery lunched with the King on board the *Victoria and Albert*, and in the afternoon, by invitation of King Edward, the Queen and Princes of Portugal visited the Royal Palace of Caserta, going by special train. On Saturday the King saw over the Carthusian Monastery and the Palace of Capodimento. His Majesty lunched later with Queen Amelia on board her pretty white yacht. An amusing incident occurred during the afternoon. As the English King and the Portuguese Queen came on deck after the meal, chatting together, a photographer was ready with his camera and succeeded in snapshotting the two sovereigns. Then hoping that he had not been observed, he turned hurriedly away, and tried to escape down the landing steps. But he had been observed, and was much alarmed at an authoritative summons to stop. He made sure that he was going to lose his precious negative, but King Edward laughingly, in French, bade him "Come nearer and take another," and the Queen of Portugal also agreed on condition that she was sent a copy. The joy of the photographer expended itself in voluble thanks and a pantomime of bows and smiles. The Neapolitans showed much enthusiasm at the gala performance at the opera on Saturday night. There was a great demonstration in Rome when the King arrived on Monday. He was met at the railway station by King Victor Emmanuel II. and the Italian Princes. The streets on the way to the Palace were superbly decorated. It is interesting to recall the fact that till this week no English Sovereign had set foot in Rome for more than a thousand years, and that it was King Ethelwulf who resided there for twelve months, having two years previously sent his five-year-old son, Alfred—afterwards the Great—to be crowned by the Pope. On Tuesday King Edward was present at a State banquet in the Quirinal, and on Wednesday he paid a visit to the Pope.

### Movements of Royalty.

QUEEN ALEXANDRA returned to England last Friday from Copenhagen, looking remarkably well. She stayed a night at Buckingham Palace and on Saturday travelled to Sandringham.

The Prince and Princess of Wales, on Saturday, unveiled the memorial erected in the Cambridge Enclosure, St. James's Park, by the officers and men of the Royal Marines to those of their comrades who lost their lives in the campaigns in South Africa and China. General French, who was present, addressed the Prince, and pointed out that on one side of the memorial was depicted the action at Graspan, where the marines helped to storm the rugged hill, and gained the highest meed of praise from Lord Methuen. On the other side was a representation of the repulse of one of the attacks by the Chinese on the Legations at Peking. Saturday was also the sixth birthday of the only daughter of the Prince and Princess of Wales, and Princess Mary of Wales was most delighted to receive a tiny Rudge-Whitworth bicycle, sent by the King to his granddaughter. A small "M" in gold is placed on the plain black enamelled frame, and the whole machine only weighs fifteen pounds. It is somewhat similar to one which his Majesty gave to Prince Edward three years ago.

The Duchess of Albany, accompanied by her daughter, Princess Alice, returned on Monday to Claremont, after four years' absence. An address of welcome was presented on the village green by the Rector, and the Duchess made a little speech expressing her pleasure at being in Esher once more.

### The Heir-Apparent and Workmen's Dwellings.

ON Monday the Prince and Princess laid the foundation-stone of a block of artizans' dwellings to be erected in the Regency Street district of Westminster. The buildings, which will accommodate 1,600 people, will cover nearly an acre and a half, and the rent of the rooms will vary from 3s. for a one-room tenement to 12s. for four-room tenements. This will include chimney sweeping and a free use of venetian blinds, baths, and hot-water supply. It is calculated that not only will the buildings be self-supporting, but that a net return on the expenditure of 3½ per cent. per annum will be able to be paid. After the ceremony the Prince and Princess attended a reception given by the Mayor and Mayoress of Westminster in the Caxton Hall.

### The War in Somaliland.

NEWS came last week from Somaliland that a serious check had been sustained by the flying column under Colonel Cobbe, near Gumburru. The column had decided to return because of shortage of water, when firing was heard in the direction of a small party of men under Captain Olivey, who had been reconnoitring westwards. Colonel Plunkett, with 160 African soldiers, and 48 Sikhs and two Maxims, were sent to assist Captain Olivey, but he met with a strong force of the enemy. After fighting till ammunition gave out, the troops formed in square and moved in the direction of Colonel Cobbe's zariba, but were ultimately overwhelmed, and all except 37 African soldiers perished, nine British officers, including Colonel Plunkett, being amongst the slain. The enemy, fighting with fanatical fury and utter contempt of death, attacked in a most determined manner, pouring in a heavy fire, and then charging, horsemen and spearmen, from all sides. Their dead lay piled up in heaps around, over 2,000 being killed. Colonel Cobbe meanwhile had so small a force that he was in a critical position, and could not move, having, moreover, only water for four days. Accordingly General Manning went to his assistance, and rescued him without any further bloodshed. At another point, Danop, a mobile column under Major Gough—who speaks highly of the troops—came in contact with the enemy, and two officers were killed and four officers and 28 rank and file wounded.

### Mr. Ritchie's First Budget.

ON Thursday of last week Mr. Ritchie, who has succeeded Sir Michael Hicks-Beach as Chancellor of the Exchequer, unfolded his first Budget in the House of Commons. During the current twelve months he estimates that he has to provide for £143,954,000. Of this, £27,000,000 would be the charge for the national debt, and would include a sinking fund of £6,600,000, against £5,750,000 in the preceding fiscal period. He computed that on the present basis of taxation the revenue would amount to £154,770,000, leaving him with a surplus of £10,816,000. This surplus is to be disposed of by taking 4d. off the income-tax, which absorbs £8,500,000, the other £2,000,000 being accounted for by the remission of the corn duty, which was only imposed last year. Mr. Ritchie proposes to move for a Committee of the House to inquire as to the incidence, machinery, and evasion of the income-tax. It is a significant fact that immediately after the introduction of the Budget, Consols advanced in price.

### For Reading to the Sick.

"THEREFORE GIVE US LOVE."

Love is like the Ocean,
Ever fresh and strong,
Birth and life and motion,
Speed and strength and song,
Which, the world surrounding, keeps it green and young.

Love is ever flowing,
Flowing ever down;
Love through all lands going,
From the Heavenly throne,
God's eternal city doth with gladness crown.

Come, thou soul that sinkest
On the desert plain,
Here of streams thou drinkest,
Ne'er to thirst again,
Which shall through thy journey feet and soul sustain.

And when this earth faileth
Love is strong as death;
Yea, o'er death prevaileth:
Love, like vital breath,
Freed from fleshly chains, the spirit cherisheth.

*Isaac Williams.*

"Love fulfils the law." Everything becomes possible to those who love. The commands of the Lord are no longer grievous, for the soul that loves is gifted by that love with fresh energies; it discovers in itself unsuspected possibilities, and is supplied with ever-flowing currents of new vigour. The impossible becomes possible to all who look to another and love; the hard loses its hardness, and the grievous ceases from grieving.—*E. B. P.*

I have spoken of what may become the increasing delight of the soul in the love of God, but we must not at all measure our love by the standard of those feelings, for "the worth of love does not consist in high feelings, but in detachment: in patience under all trials for the sake of God Whom we love."

It is not necessary that we have those high feelings, but it is necessary that we give ourselves in quiet earnestness to live the life of love, in perpetual offering of the self, rejecting all that is less than God, consecrating every desire to His glory. "Herein a man may know whether he really loves God; is he satisfied with anything less than God?" God is more to us than His gifts, even than the highest feelings of joy in Him. Seek God in love for himself alone—leave the joys to him to give or withhold; what He does is best.—*Anon.*

Help me to take up the burdens of others. Help me to know what it is to have rest in bearing an additional yoke, Thy yoke, the yoke of humanity. Help me to feel what it is to have peace in carrying a new care, Thy care, the care of universal love. Help me to learn what it is to be transfigured in the prayer for others. "The Lord turned the captivity of Job, when he prayed for his friends."—*G. Matheson.*

### Notes and Queries.

**The Editor is always willing to answer in this column, without any fee, all reasonable questions, as soon as possible.**

**But the following rules must be carefully observed:—**

**1. Every communication must be accompanied by the name and address of the writer.**

**2. The question must always bear upon nursing, directly or indirectly.**

**If an answer is required by letter a fee of half-a-crown must be enclosed with the note containing the inquiry, and we cannot undertake to forward letters addressed to correspondents making inquiries. It is therefore requested that our readers will not enclose either a stamp or a stamped envelope.**

*Medical.*

(51) Will you kindly tell me a remedy for piles inside rectum, irritable and painful at times, outside very sore through discharge. *Nurse K.*

We do not answer medical questions involving treatment. You should consult a doctor.

*Training.*

(52) Can I go as probationer at 18 years of age? If not, would a wardmaid's be a suitable place to take whilst I am under age?—*D. P.*

You are five years too young for training. We should hardly advise you to take a wardmaid's place, but all domestic arts, such as cookery, housework, needlework, etc., are useful in a nurse's education.

I am anxious to train as nurse, but I am only 4 feet 10 in height. Will you kindly tell me if this would disqualify me. I should prefer to nurse children?—*E. B.*

Your height is certainly a disadvantage, but do not let it deter you from making applications for a post, and if you fail at first perhaps you might hear, by advertising, of a matron who would accept you. State your height in the advertisement.

I shall be much obliged if you will kindly tell me if one can get trained as nurse in a London Hospital without paying a premium?—*B. M. H.*

Certainly. See the "Nursing Profession: How and Where to Train," published by The Scientific Press.

Can you tell me if there is any hospital that I could enter for general training, and where I could obtain a certificate? I am 36, and should prefer surgical cases.—*X. Y. Z.*

See the "Nursing Profession: How and Where to Train," but you are over age for training, except as a paying probationer.

*Home.*

(53) Will you kindly tell me if there is any nice home where two paralysed young women could be taken. They have about £40 a year. Up to the present they have lived with a married sister who now finds that she cannot attend to them and her own family as well.—*M. E.*

St. Mary's Home, Highmore, Parkstone, Dorset, or St. Peter's Home, Mortimer Road, Kilburn, N.W., might be suitable; or advertise.

*Male Nurse.*

(54) Will you kindly give me the name of two or three hospitals in England where I could apply for training as a male nurse. I am an Englishman and have seen some hospital work here (U.S.A.) and in China?—*H. C.*

The only hospital in England that trains male nurses is the National Hospital for the Paralysed and Epileptic, Queen's Square, Bloomsbury, W.C. Possibly you might obtain experience in prison infirmaries.

*Vegetarian.*

(55) I shall be much obliged if you can tell me of a convalescent home where vegetarian patients are received? One on the South Coast preferred.—*M. B.*

Perhaps the Secretary of the Hospital of St. Francis, New Kent Road, S.E., could give you the required information.

**Important Nursing Textbooks.**

"The Nursing Profession: How and where to Train." 2s. net; 2s. 4d. post free.

"A Handbook for Nurses." (New Edition). 5s. net; 5s. 4d. post free.

"The Human Body." 5s. post free.

"Ophthalmic Nursing." (New Edition). 3s. 6d. net; 3s. 10d. post free.

"Gynæcological Nursing." 1s. post free.

"Art of Feeding the Invalid." (Popular Edition). 1s. 6d. post free.

"Practical Hints on District Nursing." 1s. post free.

## Figures and Tables

**Fig. 1. f1:**
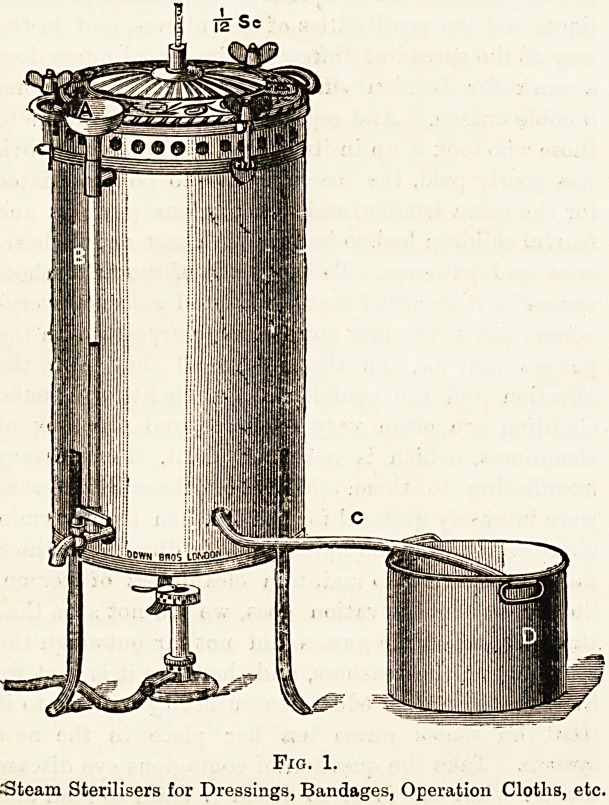


**Fig. 2. f2:**
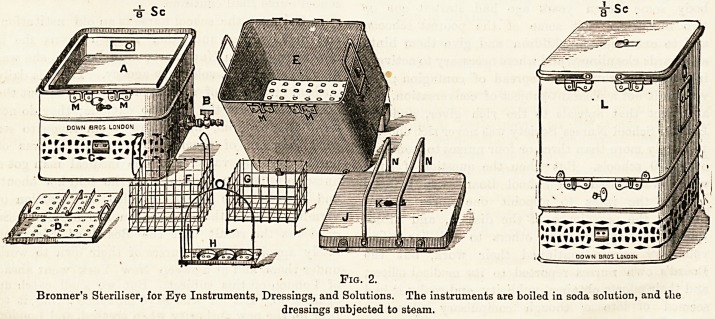


**Fig. 3. f3:**
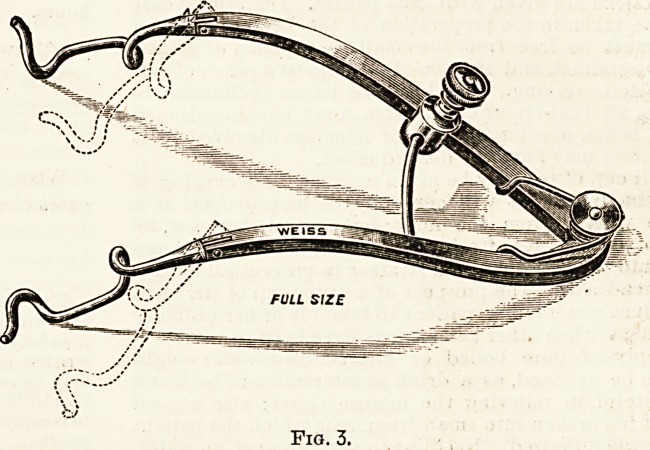


**Fig. 4. f4:**